# Mechanical Characterization of PLA+ Specimens with Different Geometries Using Experimental and Numerical Methods

**DOI:** 10.3390/polym18020243

**Published:** 2026-01-16

**Authors:** Mete Han Boztepe, Mehmet Haskul

**Affiliations:** Department of Mechanical Engineering, Şırnak University, Şırnak 73000, Türkiye; metehanboztepe@sirnak.edu.tr

**Keywords:** PLA+, additive manufacturing, fused deposition modeling, tensile testing, mechanical characterization, stress concentration, finite element analysis, geometric discontinuities

## Abstract

Geometric discontinuities are unavoidable in additively manufactured polymer components and can significantly alter their mechanical response; however, their effects are rarely quantified in a systematic and geometry-comparative manner. In this study, the tensile behavior of FDM-printed PLA+ specimens with three different geometries—dog-bone, circular-hole, and U-notched (manufactured and tested in accordance with ASTM D638 (Type IV))—was experimentally and numerically investigated. Tensile tests were conducted using a universal testing machine equipped with an extensometer, while finite element simulations were performed using an experimentally calibrated Ramberg–Osgood-based elastic–plastic material model. The dog-bone specimens exhibited an ultimate tensile strength (UTS) of 41–43 MPa and a Young’s modulus of 3.06 GPa, representing the intrinsic material response under nearly homogeneous stress conditions. Circular-hole specimens maintained comparable strength (38–42 MPa) but showed reduced ductility (1.4–1.6%) and a slightly increased apparent modulus of 3.17 GPa due to localized deformation. In contrast, U-notched specimens displayed the highest apparent modulus (≈5.30 GPa) and nominal UTS (46–49 MPa), accompanied by a pronounced reduction in ductility (0.9–1.0%), indicating severe stress concentration and predominantly brittle fracture behavior. Finite element analysis showed excellent agreement with experimental results, with peak von Mises stresses reaching approximately 42 MPa for all geometries, corresponding closely to the experimentally measured tensile strength. These results demonstrate that geometric discontinuities strongly govern stress localization, apparent stiffness, and fracture initiation in FDM-printed PLA+ components. The validated Ramberg–Osgood-based modeling framework provides a reliable tool for predicting geometry-dependent mechanical behavior under quasi-static loading and supports geometry-aware design of additively manufactured polymer structures.

## 1. Introduction

Additive manufacturing (AM) has revolutionized the production of complex polymer components across diverse industries, including aerospace, automotive, and biomedical engineering [1,2,3,4]. Among various AM techniques, fused deposition modeling (FDM) stands out as the most accessible and widely adopted method due to its cost-effectiveness, material versatility, and ease of operation [5,6]. FDM constructs three-dimensional objects through layer-by-layer extrusion of thermoplastic filaments directly from computer-aided design (CAD) models. This fabrication approach enables the production of geometrically complex structures that are challenging or economically impractical to manufacture using traditional subtractive methods [7,8].

Polylactic acid (PLA) and its enhanced variant, PLA+, are widely used in FDM applications due to their excellent processability and favorable mechanical properties. PLA+ incorporates specific additives that improve mechanical strength, toughness, and printability compared to standard PLA, making it suitable for functional prototypes and end-use parts requiring enhanced durability [9]. The mechanical performance of FDM-printed components is influenced by multiple factors, including printing parameters, material formulation, and geometric features [10,11,12].

Recent studies have shown that the mechanical and thermal performance of FDM-printed components is strongly influenced by the type of filament material used [13]. Among the most widely utilized thermoplastics, PLA and ABS represent two contrasting materials in terms of printability, strength, and environmental impact [14,15]. PLA is preferred for its biodegradability and ease of printing, while ABS offers superior toughness and thermal resistance but suffers from issues such as warping and odor emissions during processing [13,16,17,18,19,20]. A comparative summary of their fundamental properties is presented in Table 1, which highlights the inherent limitations of conventional PLA and underscores the motivation for developing advanced PLA+ formulations with improved toughness, layer adhesion, and heat stability.

Geometric discontinuities such as holes, notches, and abrupt cross-sectional changes are ubiquitous in engineering designs, serving functional purposes including assembly interfaces, weight reduction, and integration of auxiliary components. However, these features inevitably introduce stress concentrations that can significantly alter material behavior, reduce load-bearing capacity, and shift failure modes from ductile to brittle [48,49]. While stress concentration effects are well understood for conventionally manufactured materials, the anisotropic nature of FDM-printed parts—arising from layer-by-layer deposition and interlayer bonding—complicates stress redistribution and failure mechanisms, making the mechanical response of notched or perforated components less predictable [11,12].

Recent developments in 3D printing technologies (FDM) include improvements in print quality, optimized G-code strategies, and process control. The mechanical strength, surface quality, and dimensional accuracy of a part produced by FDM depend on numerous parameters such as layer thickness, infill density, raster angle, extrusion temperature, and printing speed [50,51,52,53,54,55,56]. Recent research has shown that multifactor experimental design approaches such as the Taguchi method, Response Surface Methodology (RSM), and Analysis of Variance (ANOVA) can be effectively used to optimize multiple performance parameters simultaneously in FDM 3D printing. By enabling the evaluation of parameters such as printing speed, layer thickness, infill density, and extrusion temperature, it allows researchers to identify the most influential factors on both mechanical and manufacturing efficiency. By applying such methods, significant improvements can be achieved not only in the tensile and bending properties of printed parts, but also in printing time and energy consumption [53,55,57,58,59]. Numerous studies have consistently shown that layer thickness, raster angle, and infill parameters are among the most important factors influencing the tensile and flexural strength, as well as the surface roughness, of FDM printed components. Optimizing these parameters plays a critical role in improving the mechanical integrity and dimensional accuracy of printed parts [16,17,53,57,60]. Furthermore, various post-processing techniques such as heat treatment, solvent vapor deposition, and mechanical finishing have been shown to further reduce porosity and improve surface quality, thus leading to superior structural performance and improved aesthetic properties of the final products [54,60]. Some studies emphasize that optimization in FDM 3D printing is not limited to process parameter selection but also highlight the importance of slicing and G-code algorithms. This approach aims to improve print quality, dimensional accuracy, and overall process efficiency through algorithmic enhancements rather than mere empirical adjustments [50,61].

Extensive research has demonstrated that the tensile properties of FDM-printed PLA—including ultimate tensile strength (UTS) and Young’s modulus—are strongly influenced by process parameters such as raster angle, layer height, infill density, and nozzle temperature [12,48]. Reported UTS values for PLA-based materials typically range from 20 to 80 MPa, with Young’s modulus commonly between 3 and 4 GPa, depending on processing conditions and material formulation [48,62]. Tymrak et al. [45] established baseline tensile properties for open-source RepRap systems, reporting 56.6 MPa UTS and 3368 MPa elastic modulus for PLA. Processing parameter effects have been systematically investigated by Akhoundi et al. [10], Hsueh et al. [5], Rodríguez-Panes et al. [32], and Gangwar et al. [11], demonstrating the sensitivity of mechanical performance to manufacturing conditions.

The layer-wise nature of FDM introduces significant mechanical anisotropy. Hart and Wetzel [63] examined raster angle effects on fracture behavior in ABS, demonstrating that the critical elastic-plastic strain energy release rate for intralayer fracture was approximately an order of magnitude higher than for interlayer fracture. McLouth et al. [64] investigated printing direction and raster pattern effects on fracture toughness in ABS using ASTM D5045 [65] protocols, considering three orientations (XYZ, ZXY, XZY) and two raster patterns (+45°/−45° and 0°/90°). Rankouhi et al. [66] correlated mechanical strength with layer thickness and build orientation, providing systematic data on orientation-dependent properties.

Several investigations have examined geometric discontinuity effects in additively manufactured polymers. Aliheidari et al. [67] investigated fracture resistance of FDM-fabricated ABS using Mode I opening loads on double cantilever beam (DCB) specimens, quantifying fracture energy through the J-integral method. Mishra et al. [68] examined continuous metallic fiber reinforcement effects on fracture toughness and tensile strength in recycled ABS blends, demonstrating that RABS-B/continuous brass wire composites exhibited notable enhancements compared to unreinforced materials. Senatov et al. [69] investigated the mechanical properties and shape memory behavior of 3D-printed porous PLA/hydroxyapatite scaffolds with approximately 30% porosity. While these studies provide valuable insights into fracture behavior, they focus primarily on ABS or porous structures rather than discrete stress concentrators in PLA+.

Recent work has expanded to multi-material and composite FDM systems. The adhesion mechanisms of multi-material parts were investigated by examining the influence of slicing parameters, with systematic evaluation of printing sequence, layer pattern, and infill density effects on adhesion strength for PLA–TPU, CPE–TPU, and CPE–PLA material pairs [70]. Rojas-Martínez et al. [71] developed 3D-printable PLA-based composite scaffolds reinforced with keratin and chitosan, focusing on thermomechanical performance and biological response. These studies demonstrate the expanding scope of FDM applications but do not address stress concentration in monolithic PLA+ components.

Finite element analysis (FEA) has been widely employed to predict stress distributions and deformation behavior in 3D-printed polymers [72,73]. Rodríguez-Panes et al. [32] conducted comparative FEA of PLA and ABS specimens, while Alaimo et al. [74] investigated the influence of extruded filament size and chemical composition on mechanical behavior through experimental methods. Obaidat et al. [73] developed an artificial neural network-based predictive model for tensile behavior under uncertainty. However, most computational studies validate against unnotched geometries, with limited application to stress concentration scenarios.

Most stress concentration studies examine ABS [63,64,66,67] or other polymers [68], with limited focus on PLA [69] and virtually no systematic characterization of PLA+ under stress concentration conditions. Only Grygier et al. [4] provide baseline PLA+ data without geometric discontinuities.

Existing work examines either holes, notches, or porous structures in isolation [63,64,67,68,69,71], preventing quantitative comparison across stress concentrator types. No study provides side-by-side comparison of multiple discontinuity types under identical FDM conditions. FEM studies validate primarily against unnotched specimens [32,72,73] or employ simplified constitutive models [66].

Duan et al. [75] proposed a temperature-dependent Ramberg–Osgood model for polymer-bonded composites. Sweeney et al. [76] combined biaxial testing and stress relaxation to study elastic and dissipative processes in PLA. Slavković et al. [77] experimentally investigated the thermo-mechanical behavior of 4D FDM-printed PLA under large compressive deformations. Nakai and Yokoyama [78] modeled high strain-rate compressive behavior of ABS, HDPE, PP, and PVC using a strain-rate-dependent Ramberg–Osgood equation.

Despite the extensive body of literature on the mechanical behavior of FDM-printed polymers, several critical gaps remain. Existing studies predominantly focus on process parameter optimization, printing-induced anisotropy, or unnotched specimen geometries. Investigations addressing stress concentration effects are largely limited to ABS or other thermoplastics, while PLA-based materials—particularly reinforced PLA (PLA+)—have received comparatively little attention. Moreover, studies that consider geometric discontinuities typically examine holes, notches, or porous structures in isolation, preventing direct and quantitative comparison of different stress concentrator types under identical manufacturing conditions.

In addition, most finite element investigations validate numerical models using unnotched specimens or employ simplified constitutive descriptions, offering limited insight into localized stress redistribution, plastic strain accumulation, and fracture initiation in geometrically discontinuous components. Consequently, the influence of geometric discontinuities on apparent stiffness, strain localization, and failure mechanisms in FDM-printed PLA+ remains insufficiently understood.

To address these limitations, the present study provides a systematic experimental and numerical investigation of FDM-printed PLA+ specimens with three representative geometries—dog-bone, circular-hole, and U-notched—manufactured and tested in accordance with ASTM D638 (Type IV) [79]. By maintaining identical material, printing parameters, printing orientation, and raster configuration across all specimens, the effect of geometric stress concentrators is isolated. An experimentally calibrated Ramberg–Osgood-based elastic–plastic finite element framework is employed to validate tensile test results and to elucidate geometry-driven stress localization, plastic strain distribution, and fracture initiation. This integrated approach enables a direct, geometry-aware comparison of mechanical behavior and provides design-relevant insight into the structural integrity of additively manufactured PLA+ components containing unavoidable geometric discontinuities.

## 2. Materials and Methods

### 2.1. Materials

The material used in this study was PLA+ (eSUN, Shenzhen, China), an enhanced formulation of standard polylactic acid (PLA) developed with a proprietary blend of additives to improve mechanical strength, layer adhesion, toughness, and printability. These modifications result in superior performance characteristics compared to standard PLA, making PLA+ particularly suitable for functional prototypes and end-use components requiring enhanced durability and mechanical reliability. The key mechanical and physical properties of the PLA+ filament, as provided by the manufacturer, are summarized in Table 2.

### 2.2. Design of Test Specimens

**Figure 1:**
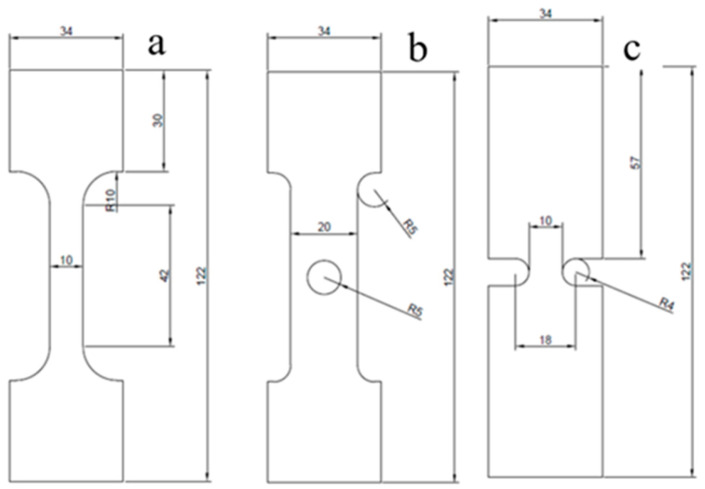
Specimen geometries and dimensions (mm): (**a**) dog-bone, (**b**) circular hole, and (**c**) U-notch configurations.

The tensile properties were characterized in accordance with the ASTM D638 standard type IV. To investigate the material’s behavior under different stress concentration conditions, specimens were fabricated in three distinct geometries: a dog-bone, a central circular hole, and a U-notch. All specimens were printed with a constant thickness of 3 mm. The overall length of each specimen was 122 mm, with a grip section width of 34 mm and a grip section length of 30 mm to ensure proper clamping during testing. The detailed dimensions and configuration of the three specimen geometries are presented in Figure 1.

### 2.3. Printing Process

**Figure 2:**
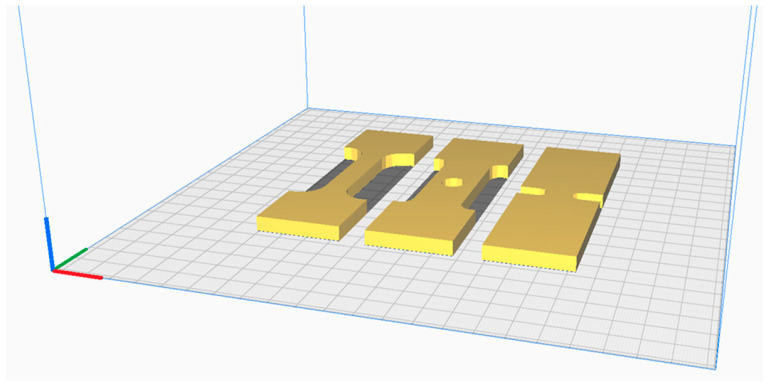
Digital layout of specimen geometries in UltiMaker Cura 5.9.0 slicing software.

The three-dimensional (3D) models of the tensile specimens were designed using CATIA (Dassault Systèmes, Vélizy-Villacoublay, France) software V5R21. Following the design phase, the models were exported in the standard tessellation language (STL) file format for additive manufacturing. These STL files were subsequently imported into the UltiMaker Cura 5.9.0 (Utrecht, The Netherlands) slicing software to generate the machine-readable G-code. The G-code was then transferred to the computer controlling the 3D printer. The digital preparation and layout of the three specimen types (dog-bone, circular hole, and U-notch) within the Cura software interface are illustrated in Figure 2.

The printing process parameters were meticulously configured in the slicing software to ensure print quality and mechanical integrity. Key parameters, including printing speed, nozzle temperature, bed temperature, infill density and pattern, and cooling fan speed, were defined. A nozzle diameter of 0.4 mm was used throughout the printing process. For this study, a nozzle temperature of 210 °C and a build plate temperature of 60 °C were maintained. A print speed of 25 mm/s was selected to enhance surface finish. Printing speed is one of the most influential parameters on the surface roughness of PLA/PLA+ parts produced by FDM; many studies have reported that the Ra value increases as the speed increases. Therefore, a printing speed of 25 mm/s was chosen to obtain a smoother surface on PLA+ samples [80,81,82,83]. A solid infill structure was achieved by setting the infill density to 100% with a grid pattern, while the cooling fan speed was set to 70% to promote adequate layer adhesion and dimensional stability.

### 2.4. Tensile Testing

**Figure 3:**
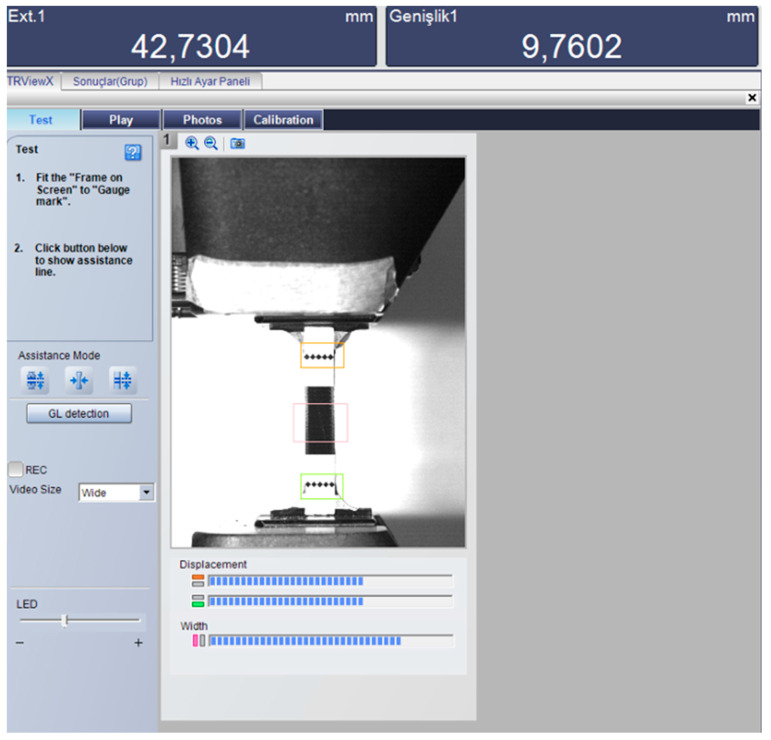
Experimental setup showing the universal testing machine with camera-based video extensometer for strain measurement.

Tensile tests were performed in accordance with the ASTM D638 standard to evaluate the mechanical properties of the PLA+ material. All experiments were conducted using a universal testing machine with a 100 kN load cell. The experimental force–displacement data reflect the force applied and the axial deformation measured by the extensometer (Figure 3). To ensure statistical reliability and repeatability of the results, three replicates of each specimen geometry were tested. The tensile tests were conducted according to ASTM D638 (Type IV) at a crosshead speed of 5 mm/min, corresponding to a strain rate of approximately 0.12 min^−1^ (1.98 × 10^−3^ s^−1^) based on a gauge length of 42 mm. For each geometry, three samples were tested to verify the reproducibility and reliability of the experimental results.

### 2.5. Finite Element Analysis

Finite element analysis (FEA) was performed using ABAQUS/Standard (Dassault Systèmes, Vélizy-Villacoublay, France) to simulate the tensile response of PLA+ specimens and to support the experimental observations. Three specimen geometries were analyzed: (i) a dog-bone specimen, (ii) a central circular hole, and (iii) a U-shaped notch. The simulations were designed to capture elastic–plastic deformation behavior, stress distributions, and stress concentration effects under quasi-static tensile loading.

No explicit fracture or damage criterion was implemented. Instead, failure initiation was assessed by correlating experimentally measured peak load conditions with localized equivalent plastic strain accumulation at stress concentrator roots, which correspond to the experimentally observed fracture initiation sites.

#### 2.5.1. Material Model and Constitutive Equations

The PLA+ material was modeled as an elastic–plastic solid using an isotropic hardening formulation based on the Ramberg–Osgood constitutive relationship. The Ramberg–Osgood model is widely employed to describe nonlinear stress–strain behavior in materials exhibiting limited plastic deformation and mild strain hardening, making it suitable for PLA-based polymers under quasi-static loading conditions.

The total strain ε is expressed as the sum of elastic and plastic components:

(1) $$\varepsilon =\varepsilon^{e}+\varepsilon^{p}$$

The elastic strain follows Hooke’s law:

(2) $$\varepsilon^{e}=\frac{\sigma }{E}$$

where *σ* is the applied stress and E is Young’s modulus.

The plastic strain component is defined using the Ramberg–Osgood formulation:

(3) $$\varepsilon^{p}={\left(\frac{\sigma }{K}\right)}^{1/n}$$

This power-law representation of the Ramberg–Osgood model, written in a form that facilitates inversion for finite element implementation, has been previously applied to polymeric materials by Nakai and Yokoyama [78].

Combining Equations (1)–(3) yields the classical Ramberg–Osgood expression for total strain:

(4) $$\varepsilon =\varepsilon^{e}+\varepsilon^{p}=\frac{\sigma }{E}+{\left(\frac{\sigma }{K}\right)}^{1/n}$$

For finite element implementation, it is more convenient to express stress as a function of plastic strain by rearranging Equation (3):

(5) $$\sigma =K{\left(\varepsilon^{p}\right)}^{n}$$

Equations (4) and (5) represent equivalent descriptions of the same constitutive behavior. In this formulation, the strain hardening exponent n characterizes the degree of work hardening, while the strength coefficient K represents the reference stress level governing the magnitude of plastic flow.

The strain hardening exponent n characterizes the material’s resistance to plastic deformation: lower values indicate limited strain hardening, whereas higher values correspond to pronounced work hardening behavior. The strength coefficient K is a reference stress parameter that scales the overall magnitude of plastic flow in the Ramberg–Osgood model. Although K is formally defined as the stress at unit plastic strain ($\varepsilon^{p}=1$), in this study it primarily functions as an estimated fitting parameter, determined to match the material behavior within the experimentally relevant plastic strain range.

#### 2.5.2. Material Parameters

The elastic properties of PLA+ were defined as follows:

- Young’s modulus, E=3061.7 MPa, determined from the linear portion of the experimental stress–strain curve,
- Poisson’s ratio, *ν* = 0.35, consistent with values commonly reported for PLA-based polymers;
- Mass density, *ρ* = 1.23 g/cm^3^ according to the manufacturer’s specification.

The Ramberg–Osgood parameters were calibrated using experimental true stress–true strain data obtained from tensile tests on standard dog-bone specimens. The plastic strain was calculated from the measured true strain by subtracting the elastic contribution:

(6) $$\varepsilon^{p}=\varepsilon^{true}-\frac{\sigma^{true}}{E}$$

Equation (5) was fitted to the experimental true stress–strain data within the strain-hardening region using an iterative parameter estimation approach. The Ramberg–Osgood parameters were determined by systematically adjusting the strength coefficient K and the strain hardening exponent n to achieve the best agreement between the model predictions and the experimental data. This fitting procedure was carried out using spreadsheet-based calculations, where the parameters were optimized based on visual comparison and minimization of deviation within the experimentally relevant strain range.

The resulting parameters were determined as follows:

- Strength coefficient, K:45 MPa
- Strain hardening exponent, n=0.014

The extremely low value of the strain hardening exponent reflects the limited plastic deformation capacity of PLA+, consistent with its quasi-brittle mechanical response and low elongation at break observed experimentally.

**Figure 4:**
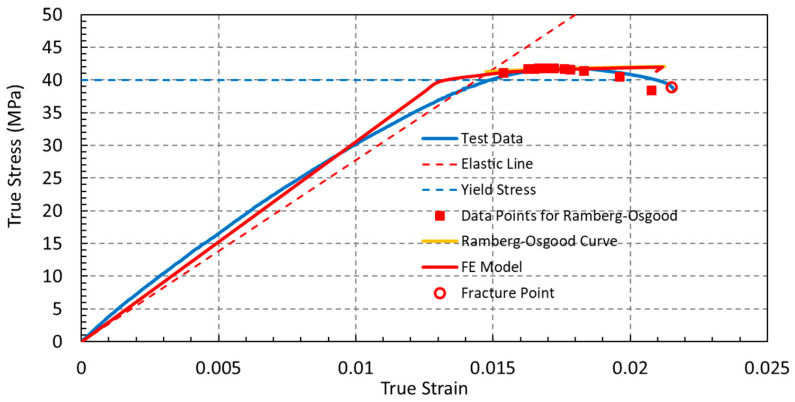
Comparison between experimental true stress–strain data, the calibrated Ramberg–Osgood constitutive model, and the finite element prediction.

The calibrated Ramberg–Osgood parameters showed very good agreement with the experimental true stress–strain data within the strain-hardening region, with the model accurately capturing the material response up to the ultimate stress (Figure 4). The low value of the strain hardening exponent (n=0.014) indicates a limited capacity for plastic deformation, which is consistent with the experimentally observed quasi-brittle failure behavior and low elongation at break (<2.5%). This response differs markedly from that of ductile metals, which typically exhibit strain hardening exponents in the range of 0.1–0.5, reflecting substantial work hardening. The elastic stress–strain response is shown in Figure 6a (E=3061.7 MPa), and Table 3 summarizes the complete material property definition used in the finite element simulations.

#### 2.5.3. Finite Element Implementation

**Figure 5:**
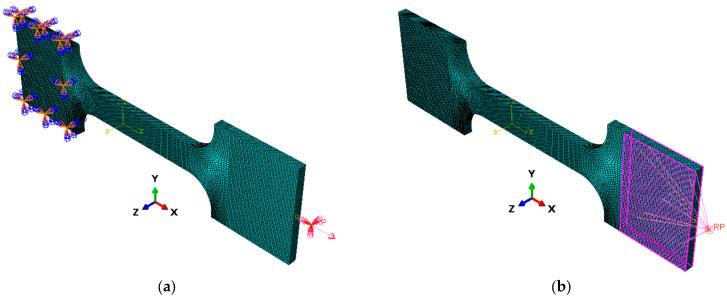
Finite element meshes and applied boundary conditions for the analyzed geometries: (**a**) Dog-bone specimen with fixed end and reference point, (**b**) Dog-bone specimen with coupling on loading surface.

Figure 5 illustrates the finite element meshes and applied boundary conditions for all specimen geometries. The nodes at the fixed end of each specimen are fully constrained to simulate the clamping effect, while displacement-controlled loading is applied at the free end through a reference point coupled to the entire end surface. This setup ensures uniform axial deformation and accurately replicates the experimental tensile test conditions.

The geometries were meshed using 8-node linear hexahedral elements (C3D8R), resulting in approximately 55,000–80,000 elements per model, depending on the geo-metric complexity. A refined mesh size of 0.5 mm was applied to all specimens.

The boundary conditions replicated the experimental setup: one end of the specimen was fully constrained in all translational degrees of freedom (U1 = U2 = U3 = 0), while a displacement corresponding to a crosshead speed of 5 mm/min was applied to the opposite end along the tensile loading direction (x-axis).

The analysis was performed using a static, general step with large deformation effects enabled (NLGEOM = ON) to account for geometric nonlinearity.

The analytical constitutive relationship given in Equation (5) was converted into a tabulated true stress–plastic strain format for finite element implementation. For selected plastic strain levels, the corresponding stress values were calculated using $\sigma =K{\left(\varepsilon^{p}\right)}^{n}$. The resulting monotonic stress–plastic strain data, covering the strain-hardening region from yield to ultimate stress, were implemented as tabular input in Abaqus (Table 3).

#### 2.5.4. Model Validation and Output

The finite element analysis outputs included von Mises stress distributions, maximum principal stress, equivalent plastic strain fields, and engineering stress–strain curves. The numerical model demonstrated very good agreement with the experimental results across all three specimen geometries. Specifically, the simulated maximum von Mises stress values, reaching approximately 42 MPa, were consistent with the experimentally determined ultimate tensile strength range of 40–49 MPa.

The predicted engineering stress-strain curves were found to capture the general trends and peak stresses of the experimental responses for dogbone, circular hole, and U-notch specimens. Overall, these comparisons support the accuracy of the finite element framework and demonstrate the suitability of calibrated Ramberg-Osgood material parameters for predicting stress distributions and deformation behavior under quasi-static loading conditions.

The predicted engineering stress–strain curves were found to capture the general trends and peak stresses of the experimental responses for the dog-bone, circular-hole, and U-notched specimens. Overall, these comparisons support the fidelity of the finite element framework and demonstrate the suitability of the calibrated Ramberg–Osgood material parameters for predicting stress distributions and deformation behavior under quasi-static loading conditions.

#### 2.5.5. Physical Interpretation

The extremely low strain hardening exponent (n=0.014) indicates a very limited strain-hardening capacity, which is characteristic of brittle or quasi-brittle materials. This finding is consistent with the experimentally measured low (<2.5%), the rapid transition from elastic deformation to fracture, and predominantly brittle failure mode observed in the tensile tests.

## 3. Results

The mechanical properties of PLA+ were investigated using a combination of experimental tensile tests and finite element analysis (FEA) on three specimen geometries: a dog-bone, a circular hole, and a U-notch. This integrated approach provides comprehensive insights into the material’s elastic-plastic behavior, strength limits, and deformation characteristics under various stress conditions.

### 3.1. Tensile Properties and Apparent Elastic Modulus for Different Geometries

**Figure 6:**
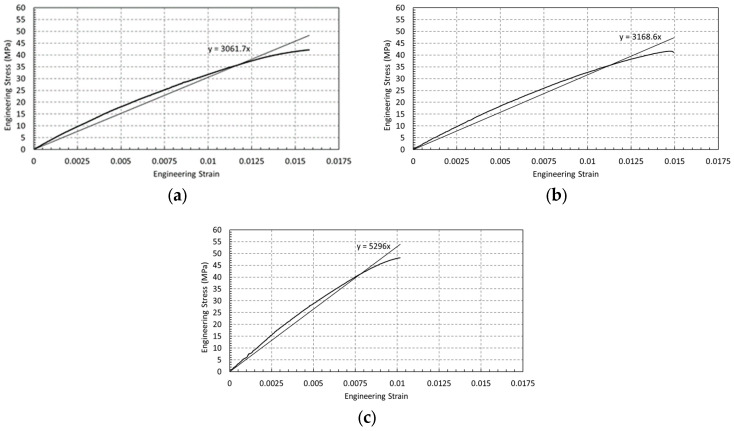
Elastic region and linear fit for (**a**) dog-bone (**b**) circular hole (**c**) U-notch specimens (E value annotated in the graph).

The elastic response of FDM-printed PLA+ specimens was evaluated from the initial linear region of the engineering stress–strain curves for dog-bone, circular-hole, and U-notched geometries (Figure 6a–c). For specimens with geometric discontinuities, the reported elastic modulus is an apparent modulus, since the measured strain reflects geometry-induced nonuniform deformation rather than the intrinsic material behavior. Linear regression of the elastic region yielded geometry-dependent stiffness values of 3.06 GPa (dog-bone), 3.17 GPa (circular-hole), and 5.30 GPa (U-notch). The nearly identical elastic slopes observed for the dog-bone and circular-hole specimens indicate that the intrinsic elastic response of PLA+ is largely preserved under uniform or mildly perturbed stress states, despite the presence of a circular discontinuity.

In contrast, the significantly higher apparent elastic modulus measured for the U-notched specimens (Figure 6c) does not represent an actual increase in material stiffness. Instead, it arises from localized strain underestimation near the notch root, where triaxial stress states and geometric constraints suppress global strain measurements. Consequently, the calculated modulus reflects the influence of geometric constraint on deformation rather than a true change in the elastic properties of PLA+. These results demonstrate that geometric discontinuities can strongly bias experimentally measured stiffness in FDM-printed components, highlighting the necessity of distinguishing between intrinsic material behavior and geometry-dependent apparent responses.

### 3.2. Stress and Plastic Strain Distributions

**Figure 7:**
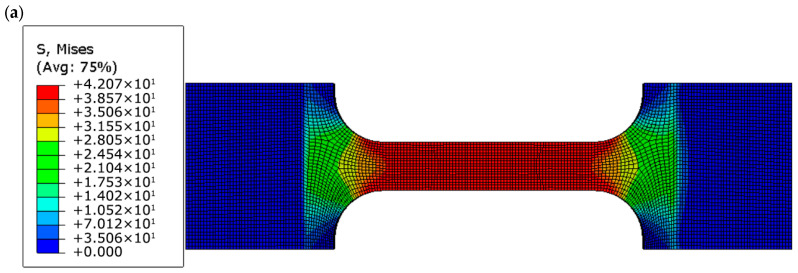

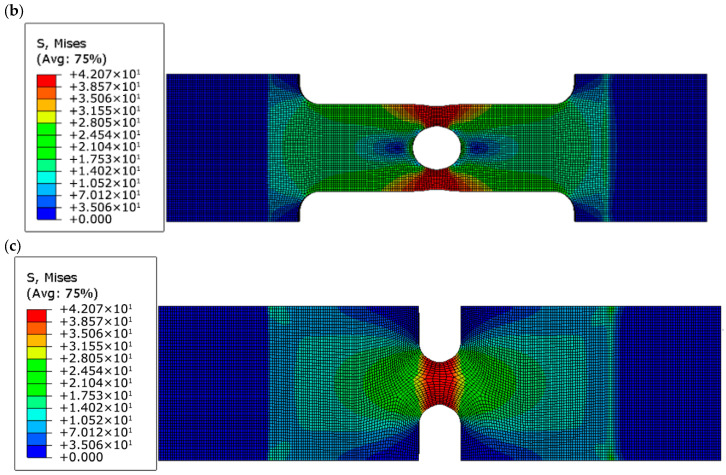
von Mises stress distributions at peak load for (**a**) dog-bone, (**b**) circular-hole, and (**c**) U-notched PLA+ specimens, highlighting stress concentration at geometric discontinuities.

**Figure 8:**
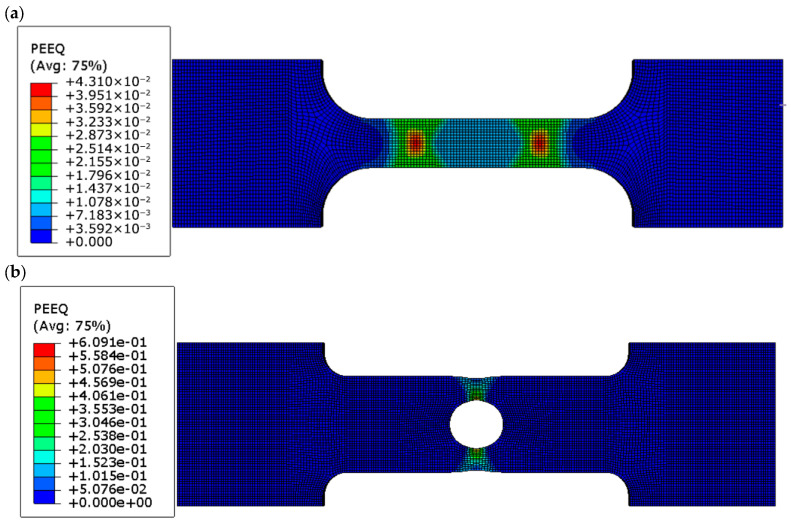

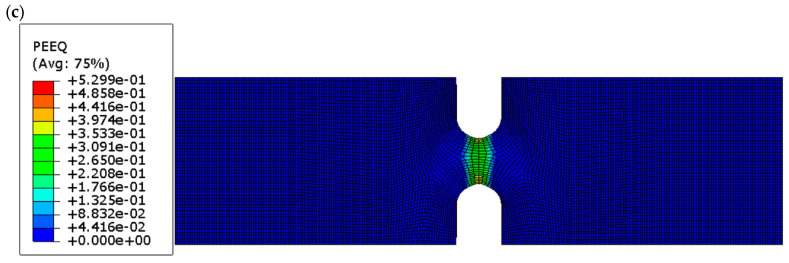
Equivalent plastic strain (PEEQ) distributions at peak load for (**a**) dog-bone, (**b**) circular-hole, and (**c**) U-notched PLA+ specimens, showing localized plastic deformation near stress concentrator roots.

To examine the influence of geometric discontinuities on the mechanical response of FDM-printed PLA+ specimens, finite element analyses were performed to evaluate the distributions of von Mises stress (Figure 7a–c) and equivalent plastic strain (PEEQ) (Figure 8a–c) at peak load. These results provide insight into geometry-induced stress localization and the initiation of localized plastic deformation across the different specimen geometries.

Both the circular-hole and U-notched specimens exhibit pronounced localization of equivalent plastic strain near the geometric discontinuities. This localization results from the combined effects of stress concentration, elevated stress triaxiality, and displacement-controlled loading. The high local PEEQ values indicate strain localization within a confined region, reflecting the initiation of failure rather than uniform plastic deformation throughout the specimen. Consequently, the PEEQ contours are interpreted qualitatively to assess the spatial distribution of plastic deformation and to identify potential fracture initiation sites.

### 3.3. Experimental–Numerical Correlation and Effect of Specimen Geometry

**Figure 9:**
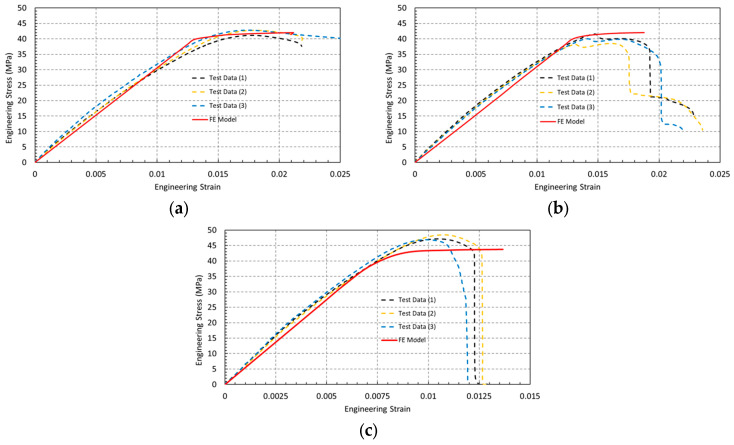
Engineering stress–strain curves for (**a**) dog-bone specimens, (**b**) circular-hole specimens, and (**c**) U-notch specimens, showing a comparison between experimental results (three replicates) and finite element simulations.

Figure 9 presents a comparison between the engineering stress–strain curves obtained from experimental tensile tests and finite element (FE) simulations for the three specimen geometries, namely dog-bone, circular-hole, and U-notch specimens. For all configurations, three experimental replicates are shown, demonstrating good repeatability and consistency of the manufacturing process and testing conditions. Overall, a close agreement between the experimental results and FE predictions is observed up to the ultimate tensile strength, confirming the reliability of the numerical model.

Dog-bone specimens (Figure 9a) exhibit a well-defined linear elastic region followed by yielding and limited plastic deformation prior to fracture. The strain at ultimate tensile strength ranges between 1.7% and 1.8%, while the strain at rupture varies from 2.1% to 2.4%, as summarized in Table 4. These relatively low strain values indicate that, despite the improved strength of PLA+ compared to conventional PLA, the material retains a predominantly brittle response under uniaxial tensile loading. The FE model accurately reproduces the elastic modulus, ultimate tensile strength, and corresponding strain values, validating the adopted elastic–plastic constitutive law.

Circular-hole specimens (Figure 9b) show a similar elastic response but a reduced elongation at maximum stress compared to the dog-bone geometry. The ultimate tensile strength ranges between approximately 38 and 42 MPa, with elongation at maximum stress between 1.4% and 1.6% (Table 4). While the FE simulations accurately capture the elastic regime and the stress level at the onset of failure, the abrupt post-peak stress drop observed experimentally is not reproduced, as the numerical model does not incorporate an explicit damage or fracture criterion.

U-notch specimens (Figure 9c) exhibit a steep linear elastic response followed by an abrupt stress drop immediately after reaching the ultimate tensile strength, indicating a predominantly brittle failure mode with very limited plastic deformation. The ultimate tensile strength is reached at a low engineering strain of approximately 0.9–1.0% (Table 4), highlighting the strong notch sensitivity of PLA+. Although the nominal strength appears higher due to the reduced load-bearing cross-section, failure initiates once the local stress at the notch root approaches the intrinsic material strength.

The corresponding von Mises stress distributions obtained from the FE analyses further support these observations. In all cases, the maximum von Mises stress reaches approximately 42 MPa, in close agreement with the experimentally measured ultimate tensile strength. Stress localization is observed in the gauge section of the dog-bone specimens, at the edge of the circular hole, and at the root of the U-notch, confirming that fracture initiation is governed by local stress concentration rather than nominal applied stress.

**Figure 10:**
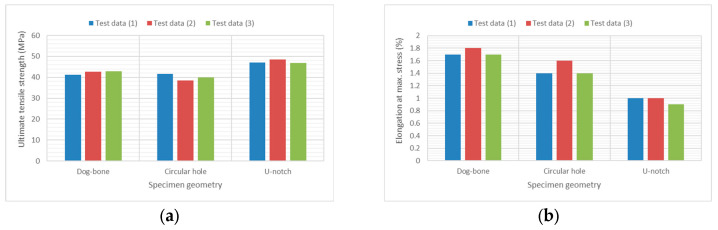
Column charts comparing (**a**) ultimate tensile strength and (**b**) elongation at maximum stress for dog-bone, circular-hole, and U-notch specimens.

Table 4 summarizes the ultimate tensile strength and engineering strain at maximum stress for the three specimen geometries. To facilitate visual comparison, the corresponding column charts are shown in Figure 10.

## 4. Discussion

This study demonstrates that geometric discontinuities significantly influence the mechanical response of FDM-printed PLA+ components, affecting ductility, failure mode, and the experimentally measured elastic modulus. Dog-bone specimens exhibited a balanced response with ultimate tensile strength (UTS) values of 41–43 MPa, engineering strain at maximum stress of 1.7–1.8%, and an elastic modulus of approximately 3.1 GPa, representing the baseline mechanical behavior under nearly uniform stress and strain conditions. In contrast, specimens containing a central circular hole maintained similar UTS values (38–42 MPa) but showed a noticeable reduction in ductility (1.4–1.6%) and a slightly altered apparent modulus (~3.17 GPa), reflecting pronounced stress and strain localization at the hole edge. The U-notched specimens exhibited the most severe response: although nominal UTS appeared higher (46–49 MPa) due to reduced effective cross-sectional area, engineering strain at maximum stress dropped sharply to 0.9–1.0%, and apparent modulus increased significantly (~5.3 GPa), highlighting the impact of high stress triaxiality and geometric constraint on local deformation.

Finite element (FE) analysis corroborated these experimental observations, reveal-ing maximum von Mises stresses of approximately 42 MPa in all geometries, confirming that fracture initiation is governed by intrinsic material strength at stress concentration sites. The Ramberg–Osgood constitutive model successfully captured the elastic–plastic transition in dog-bone specimens, while stress concentrators primarily dictated local failure in notched geometries. Equivalent plastic strain (PEEQ) analysis further emphasized the influence of geometry, showing relatively homogeneous deformation in dog-bone specimens (~0.043) and intense localization in circular-hole (~0.61) and U-notch (~0.53) specimens, explaining abrupt post-peak stress drops and identifying probable fracture initiation sites. These results underscore the critical role of geometric features in governing apparent stiffness, ductility, and nominal versus local stress interpretations. From a design perspective, sharp notches and abrupt cross-sectional changes should be avoided or mitigated through fillets, gradual transitions, or local reinforcement, ensuring reliable structural performance of FDM-printed PLA+ components.

## 5. Conclusions

This study systematically investigated the influence of geometric discontinuities on the mechanical response of FDM-printed PLA+ components through combined experimental tensile testing and finite element analysis. Based on the obtained results, the following conclusions can be drawn:

- Baseline mechanical behavior:

Unnotched dog-bone specimens exhibited a balanced mechanical response, with an ultimate tensile strength of 41–43 MPa, an elastic modulus of approximately 3.1 GPa, and elongation at maximum stress of 1.7–1.8%. These values represent the intrinsic tensile behavior of PLA+ under nearly homogeneous stress and strain conditions.

- Effect of circular holes:

The introduction of a central circular hole preserved the ultimate tensile strength (38–42 MPa) but reduced ductility to 1.4–1.6%. Finite element results revealed pronounced stress and strain localization at the hole edge, demonstrating that ductility degradation precedes strength loss when moderate geometric stress concentrators are present.

- Effect of U-notches:

U-notched specimens exhibited an apparent increase in nominal ultimate tensile strength (46–49 MPa); however, this effect is purely geometric and results from the reduced effective load-bearing cross-sectional area. The sharp reduction in elongation to 0.9–1.0% confirms the high notch sensitivity of PLA+. Severe stress triaxiality at the notch root suppresses plastic deformation, leading to premature brittle fracture.

- Experimental–numerical correlation:

Finite element simulations showed excellent agreement with experimental results, accurately predicting stress distributions, ultimate load levels, and fracture initiation sites. In all investigated geometries, fracture initiation occurred when the local von Mises stress reached approximately 42 MPa, indicating that failure is governed by the intrinsic strength of PLA+ rather than nominal applied stress. The validated Ramberg–Osgood constitutive model provides a reliable framework for analyzing homogeneous and geometrically discontinuous PLA+ structures.

- Design implications:

The results demonstrate that geometric discontinuities strongly influence not only strength and ductility but also the apparent elastic modulus obtained from experimental measurements. Sharp notches and abrupt cross-sectional changes should therefore be avoided or mitigated through design strategies such as fillets, gradual transitions, or local reinforcement. Geometry-aware design and careful interpretation of mechanical test data are essential for the safe and reliable structural application of FDM-printed PLA+ components.

Overall, the integration of experimental characterization with validated finite element modeling established in this study provides a robust methodology for evaluating and optimizing the mechanical performance of additively manufactured polymer components. Future work will focus on the combined effects of printing-induced anisotropy, build orientation, and stress concentrator severity to further expand the design envelope of FDM-printed PLA+ structures.

## Figures and Tables

**Table 1:** Comparison of typical properties of PLA and ABS used in FDM 3D printing.

Property	PLA	ABS	Key References
Chemical nature	Polylactic acid (biodegradable polyester)	Acrylonitrile–butadiene–styrene (petroleum-based polymer)	[17,18,21,22,23,24]
Print temperature (°C)	190–220	230–270	[18,25]
Glass transition temperature (°C)	~60–65	~105	[26,27,28]
Tensile strength (MPa)	50–70	30–50	[18,25,29]
Elongation at break (%)	2–10	20–40	[18,30,31,32]
Density (g/cm^3^)	~1.25	~1.05	[25,32,33]
Impact resistance	Low	Moderate to high	[30,31,32,34,35,36]
Thermal resistance (°C)	Poor (~50–60)	Better (~70–80)	[13,37,38]
Biodegradability	High	Non-biodegradable	[38,39,40,41,42,43,44]
Typical issues in FDM printing	Brittle behavior, low heat resistance	Warping, odor emission	[13,19,20,39,45,46,47]
Need for improvement	Improved PLA+	-	Present study

**Table 2:** Mechanical and Physical Properties of PLA+ Material.

Density (g/cm^3^)	1.23
Tensile Strength (MPa)	60
Flexural Strength (MPa)	74
Flexural Modulus (MPa)	1973
Elongation at Break (%)	20
Extruder Temperature (°C)	210–230
Bed Temperature (°C)	45–60
Printing Speed (mm/s)	40–100
Layer Thickness (mm)	0.2
Printer Resolution (mm)	0.2

**Table 3:** Material Properties for Finite Element Model.

Elastic Properties			
Property	Symbol	Value	Unit
Young’s Modulus	*E*	3061.7	MPa
Poisson’s Ratio	ν	0.35	-
Density	*ρ*	1.23	g/cm3
$\mathrm{Plastic}\;\mathrm{Properties}\;(\mathrm{Ramberg}–\mathrm{Osgood}:\;\sigma =K{\left(\varepsilon^{p}\right)}^{n}$)			
Property	Symbol	Value	Unit
Strength Coefficient	*K*	45	MPa
Strain Hardening Exponent	*n*	0.014	-
Tabular Plastic Data For Abaqus (Hardening Region)			
True Stress (MPa)		Plastic Strain	
41.11		0.00263	
41.67		0.00335	
41.73		0.00345	
41.79		0.00369	
41.83		0.00398	
41.73		0.00465	
41.64		0.00490	
41.40		0.00545	
40.53		0.00703	
38.48		0.00882	

**Table 4:** Ultimate tensile strength and elongation at maximum stress for different specimen geometries.

Specimen Type	Samples	Ultimate Tensile Strength (MPa)	Elongation at Max. Stress (%)
Dog-bone	1	41.13	1.7
Dog-bone	2	42.75	1.8
Dog-bone	3	42.80	1.7
Circular hole	1	41.56	1.4
Circular hole	2	38.50	1.6
Circular hole	3	40.02	1.4
U-notch	1	47.15	1.0
U-notch	2	48.49	1.0
U-notch	3	46.92	0.9

## Data Availability

The original contributions presented in this study are included in the article. Further inquiries can be directed to the corresponding author.
